# Balance Assessment on a Modified Posturomed Platform in Healthy Dogs

**DOI:** 10.3390/vetsci11100498

**Published:** 2024-10-12

**Authors:** Viola Wolszky, Yury Zablotski, Andrea Fischer, Susanne Lauer

**Affiliations:** 1 LMU Small Animal Clinic, Centre for Clinical Veterinary Medicine, Ludwig-Maximilians-University Munich, 80539 Munich, Germanyy.zablotski@med.vetmed.uni-muenchen.de (Y.Z.);; 2 Small Animal Department, Faculty of Veterinary Medicine, Leipzig University, 04103 Leipzig, Germany

**Keywords:** canine, Posturomed, balance platform, posturography, center of pressure

## Abstract

**Simple Summary:**

The reliability of a modified Posturomed balance platform was assessed for static and dynamic posturographic measurements in healthy dogs at two different time points. Static posturographic measurements appeared reliable, while results indicated a slight to moderate training effect for two of the three tested center of pressure parameters during both dynamic conditions. Further studies are needed to investigate Posturomed-based balance analysis in dogs with neurologic and orthopedic diseases.

**Abstract:**

Reliable, standardized balance tests for dogs are not available yet. The purpose of this study was to investigate the reliability of static and dynamic posturography in healthy dogs. Healthy dogs (*n* = 20) were positioned with four paws longitudinally and with the forepaws only transversely on a modified pressure-sensitive balance platform (Posturomed-FDM-JS, Zebris, Isny, Germany). Three static and dynamic posturographic trials were recorded (recording duration: 20 s) and repeated after 7–14 days. Center of pressure (COP) parameters COP-path-length (PL; mm), 95% COP-confidence-ellipse-area (CEA; mm^2^), and COP-average-velocity (AV; mm/s) were calculated for the first steady-state 5 s intervals of each trial. The reliability of COP parameters was assessed with robust linear mixed effects models with nested random effects of patient and trial. The training effect was analyzed using Cohen’s d. For static posturography, PL, CEA, and AV did not differ significantly between time points; CEA had the highest reliability (*p* = 0.92). For dynamic posturography, AV and PL differed significantly between time points (AV: *p* ≤ 0.043; PL: *p* ≤ 0.045). Slight training effects were observed for transverse positioning (Cohen’s d: PL 0.65; AV 0.267) and moderate training effects for longitudinal positioning (Cohen’s d: PL: 0.772; AV: 0.783). This study showed that static posturography on a modified Posturomed-balance platform was reliable in healthy dogs but indicated training effects during dynamic posturography.

## 1. Introduction

Balance requires a coordinated complex interaction between the visual, auditory, vestibular, motor, and higher-level premotor systems to maintain postural and equilibrium control [1,2,3].

Throughout a quiet stance, postural sway due to swaying movements of the body arises from reactive movements needed to counteract destabilizing forces to prevent loss of balance [4,5]. Balance impairment due to neurologic or orthopedic disorders, age, or inadequate training frequently correlates with an increased risk of injury and/or decreased functional performance during daily life in people. Thus, balance training and assessment not only play an important role in injury prevention and prediction but also in human sports medicine [6,7,8]. Principally, clinical balance tests can be assigned to three major categories: (1) Functional assessment, (2) system-dependent physiological assessment, and (3) quantitative assessment [9].

In people, functional assessments in the form of “battery tests” (for example, the Tinetti test, Berg Balance Scale, or Timed Up and Go Test) have the advantage of being applicable with minimal training and equipment in a short time in the field [10,11,12]. During battery tests, observers time and score the performance of individuals throughout a set of motor tasks to assess their balance abilities. Although functional assessment tests frequently allow for the prediction of risk for falls and subsequent need for therapy in people, they have the disadvantage of not detecting subtle changes occurring throughout therapy. Moreover, functional assessments frequently have a ceiling effect [13] and do not allow us to determine the ultimate cause for the balance deficit [7,9,14]. In veterinary medicine, there is a relative paucity with regard to validated functional balance assessment tests, and comparative population data are currently still missing. Hemistanding and walking and observation of balancing maneuvers, while equilibrium is manually challenged by the observer, are crude basic static and dynamic tests that are commonly utilized in practice [15]. Recently, functional battery tests have been developed to evaluate overall fitness in working dogs and to score motor function in dogs recovering from neurologic disorders, but balance is still primarily assessed indirectly by observing simple task performance [16,17,18,19].

System-dependent physiological assessments (for example, the Balance Evaluation Systems Test (BESTest) or Physiological Balance Profile (PPA)) are designed with the goal of identifying the underlying cause or mechanism for human balance disorders, mainly investigating different physiologic balance control systems [20,21]. However, this type of assessment has not been established yet in veterinary medicine.

Static and dynamic posturography and wearable motion sensor systems have been employed for objective quantitative assessment of balance performance in human medicine, measuring center of pressure (COP) variability [9,22,23]. Compared to clinical, functional assessments, computer-controlled posturographic measurement systems have been shown to be more sensitive to diagnosing balance disorders and predicting falls related to age, stroke, or multiple sclerosis [24,25,26,27]. During static posturography, people stand as still as possible, while postural sway is determined by measuring displacements of the center of foot pressure on a force plate/pressure mat or trunk or head movements with accelerometers or gyros [9,23]. Static posturography has the ability to detect balance impairments and increased risk for injury in patients with neurologic diseases like multiple sclerosis but is not sensitive enough to detect subtle changes in athletes [27,28]. Although human static posturographic tests can be modified to increase demands on the postural control system (altering of surface, positioning, visual input, or by add-on of secondary tasks), posturography under dynamic conditions is considered more crucial when assessing the balancing abilities of athletic individuals [29]. During dynamic posturography, balance is typically challenged, inducing sudden horizontal or rotatory perturbation to instrumented computerized movable support platforms or treadmill systems to test the anticipatory and compensatory balance systems [9]. When perturbations occur during stance in people, postural sway increases, while reactive compensatory measures strongly depend on the type of perturbation [28]. In human medicine, dynamic posturographic platforms like Posturomed (Haider Bioswing, Pullenreuth, Germany) or Wii Balance Board (WBB) (Nintendo, Kyoto, Japan) have been shown to allow for reliable and reproducible balance assessment [27,30,31].

Quantitative balance analysis is still a relatively new field in veterinary medicine. In standing horses, static posturography was performed to assess the effect of blindfolding on COP variability [32]. Body COP could be measured and referenced from an anatomical marker in healthy dogs and dogs with neurologic deficits walking on an instrumented treadmill with fair to excellent reliability [33,34]. Paw and body COP parameters have been measured in healthy dogs and were compared to dogs with cranial cruciate ligament disease and elbow and hip osteoarthritis standing or walking over pressure-sensitive platforms or walkways [35,36,37,38,39,40]. Age-related effects on COP parameters were recently investigated by comparing geriatric to healthy adult dogs, indicating that in dogs, postural sway increases with age and is highly affected by joint pain [41]. Recently, external perturbation induced by a motorized balance board (Imoove-vet^®^ platform, Allcare Innovations, 26500 Bourg les Valence, France) was shown to challenge postural stability in healthy dogs with an increase in platform amplitude, having a greater effect on COP parameters than the increase in speed [42].

To the authors’ knowledge, static and dynamic posturography on a Posturomed balance platform has not been reported in dogs. The goal of this study was to evaluate the reproducibility and reliability of a modified Posturomed platform as a quantitative balance measurement device for dogs.

We hypothesized that the balance ability of healthy dogs can be measured reliably with high reproducibility on a modified Posturomed platform.

## 2. Materials and Methods

### 2.1. Dogs

Twenty healthy client-owned adult dogs were enrolled in this prospective controlled trial. Dogs were considered eligible for the study if they had a body weight of 10–40 kg, were older than one year, and did not have any previous history of orthopedic or neurologic disorders.

Only dogs without evidence of musculoskeletal abnormalities during physical examination and upon objective gait analysis were included in this study. Dogs were excluded if they were too large to stand completely on the balance platform in the longitudinal direction. Written informed owner consent was obtained for each dog. All dogs were handled in accordance with a protocol approved by the institutional ethics committee (Nr. 258-01-03-2021, date of approval 7 June 2021).

The signalment (breed, age, weight, sex, and body condition score) of each dog was documented. Upon entry of the study, all dogs underwent complete general, orthopedic, and neurologic examinations. Objective gait analysis with dogs walking and trotting on a pressure-sensitive treadmill (CanidGait^®^, zebris Medical GmbH, Isny, Germany) and static and dynamic balance analysis were performed on the initial day of screening and were repeated after 7–14 days.

### 2.2. Canine Posturomed Balance Platform Prototype

**Figure 1:**
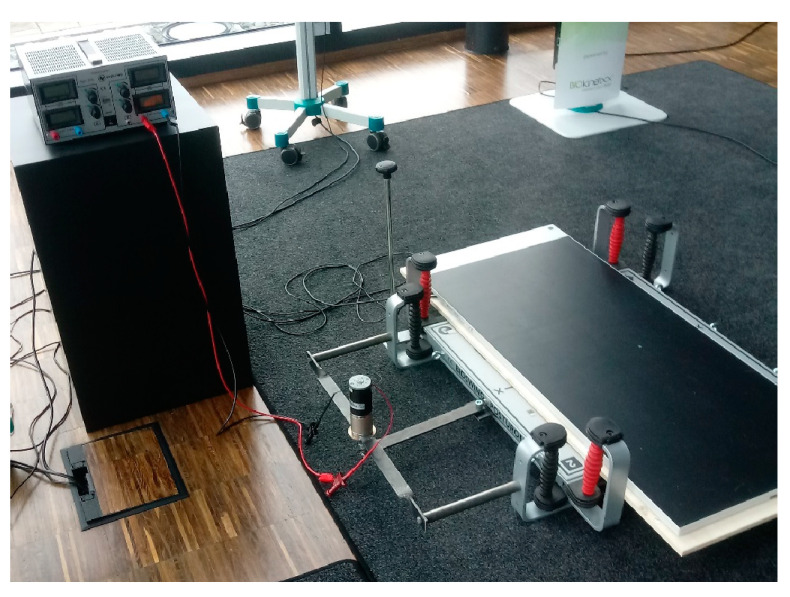
Modified canine Posturomed platform.

The Posturomed balance platform (external dimensions: 60 cm × 60 cm; Haider Bioswing GmbH, Pullenreuth, Germany) is hung up on steel cables with a plastic casing for damping, allowing for free swinging of the platform. Unlocking of the platform allows for undulations in the horizontal plane. For the prototype, the hand railing was removed from a standard Posturomed balance platform. A custom-made pressure-sensitive distribution measuring plate (external dimensions: 105.5 cm × 44.8 cm × 2.1 cm; measuring surface: 94.8 cm × 40.6 cm with 5376 integrated pressure sensors; Zebris FDM-JS System, zebris Medical GmbH, Isny, Germany) was firmly attached with double-sided tape to the surface of the swinging platform (Figure 1). The standing area was divided into four equal quadrants with elastic tape. The original lever-based provocation unit was replaced with a deflection mechanism that is regulated with a stepless gear motor moving the platform horizontally by 1.5 cm to the side (x-direction). A transformer allowed for continuous regulation of the platform deflection frequency. The target frequency of the horizontal deflection was set at 1.2 deflections per second.

Video sequences were recorded with two cameras (SYNCLightCam, zebris Medical GmbH, Isny, Germany) synchronized with the FDM-JS system (WinFDM Software v1.2.2; zebris Medical GmbH, Isny, Germany; sampling rate: 100 Hz). One camera was positioned behind, and one camera was on the right side of the platform.

### 2.3. Static and Dynamic Balance Analysis

Acclimatization—Balance analysis was executed in a quiet room. After an initial acclimatization period of up to 15 min (depending on the dog’s behavior), each dog was walked on a leash three times in a longitudinal direction (y-direction) over the balance platform to become familiar with the set-up. After this acclimatization period, one test measurement was performed under static conditions with the dog standing in the y-direction on the unlocked platform.

**Figure 2:**
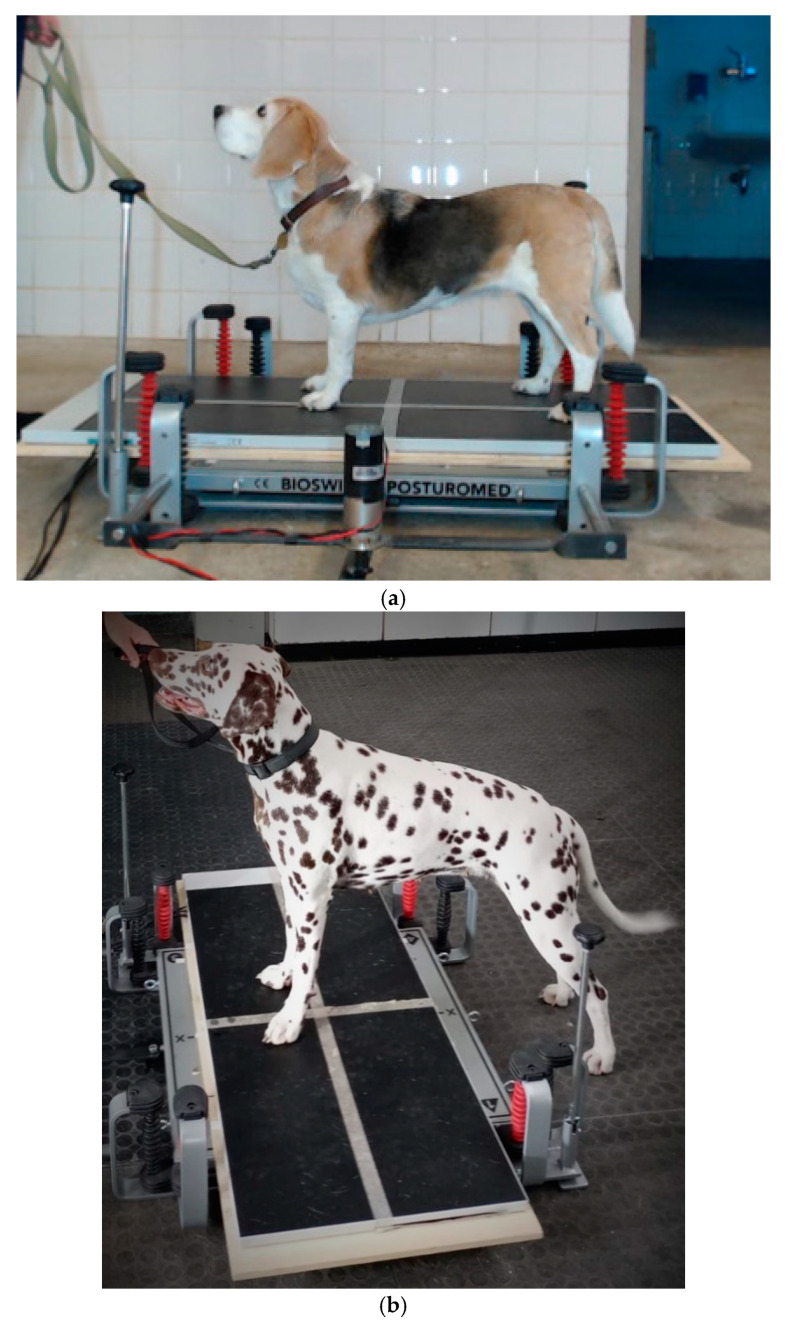
(**a**): Standing position in the longitudinal y-direction. The paws are oriented to be in each predetermined field to ensure as straight standing as possible. (**b**): Standing position in the transverse x-direction. Only the forepaws are placed on the platform; the hindpaws stand on the ground opposite to the deflection mechanism.

Balance measurements—Each dog was positioned three times under static conditions and then three times under dynamic conditions in the y-direction (Dy) on the platform (Figure 2a). Finally, the dog was positioned under dynamic conditions transversely in the x-direction (Dx), with the forepaws only on the platform facing the deflection mechanism (Figure 2b).

Each dog was primarily handled by one single person (the dog owner) for each trial at both time points. All handlers were instructed and supported by the first author. The primary handler was standing in front of the dog and directed the dog verbally or with treats to stand as straight and symmetric as possible and without head movement on the platform. As soon as the dog remained steady and calm on the platform, a 20 s long posturographic measurement was executed. Body contact of the handler with the dog or pulling on the leash was minimized as much as possible. All dogs left the balance platform after each trial and were then repositioned on the platform for the next measurement.

For trials performed under dynamic conditions, the transformer was gradually upregulated with the dog on the platform until the target frequency of 1.2 horizontal deflections per second (70–74 deflections per minute based on dummy trials) was reached. Upregulation time was based on the comfort level of the dog. Dogs were also allowed to jump directly on the already dynamized platform at target frequency. Overall evaluation time was limited to 30 min.

### 2.4. Data Analysis

Videos of each balance measurement were reviewed by one investigator (VW) to identify sequences with the dog standing most symmetric with minimal movement of body and head. For a valid balance measurement, the dog had to hold this position for ≥5 s without corrective stepping. Three valid measurements recorded separately for each condition were required for a successful trial.

The pressure distribution of all limbs (measured in N/cm^2^ and expressed as a percentage of the body weight) was measured with a sampling rate of 100 Hz. The center of pressure (COP) was calculated individually for the forelimbs and the hindlimbs and for the entire body. The COP variability was measured in perpendicular x and y directions. The 95% COP confidence ellipse (in mm^2^), the COP path length (in mm), and the COP average velocity (in mm/s) were calculated by the proprietary software (Animal Analysis Suite RC, Version 2.3.24, zebris Medical GmbH, Isny, Germany). Video recordings of each trial were recorded synchronously to the COP measurements with the dogs positioned on the balance platform. The 95% COP confidence ellipse area (CEA) was defined as the area that included 95% of all individual measured COPs within one measurement. COP path length (PL) was defined as the total length of the COP sway. COP average velocity (AV) was defined as the velocity within the COP sway.

### 2.5. Statistical Analysis

R statistical software (R version 4.4.1 (2024-06-14)) was used for data analysis. Descriptive statistics were determined regarding weight, age, and shoulder height. The normality of data distribution was evaluated with the Shapiro–Wilk test. Differences between the two examination days were identified. Robust linear mixed-effects models with the nested random effects of patient and trial on the intercept were performed to compare the first and second appointments of area, length, and speed for the three conditions (S, Dy, and Dx). Values of Cohen’s d (effect size) were calculated to determine a more interpretable effect size of the COP parameters (CEA, PL, and AV) for the three conditions (S, Dy, and Dx). An effect size < 0.2 was regarded as negligible, from 0.2 to 0.5 as slight, 0.5 to 0.8 as moderate, and >0.8 as a clear effect. The Tukey method was used to correct the *p*-values for multiple testing. Values of *p* ≤ 0.05 were considered significant. CEA, PL, and AV were studied for the three conditions (S, Dy, and Dx) via generalized linear mixed-effect models with a gamma distribution family.

## 3. Results

### 3.1. Dogs

The study population consisted of six mixed breed dogs, two Dalmatians, and one of each of the following breeds: German Shepherd Podenco, Labrador, Doberman Pinscher, English Springer Spaniel, Rhodesian Ridgeback, Border Collie, Beagle, Hunting Terrier, Dogo Argentino-Mix, Keeshond, Bretone, and Australian Shepherd. Six dogs were males, and fourteen were females, of which ten were spayed females, five were neutered males, four dogs were intact females, and one was a sexually intact male. The dogs had a mean age of 5.4 ± 3.3 years. The mean shoulder height was 52.0 ± 8.9 cm. The mean body weight was 20.7 ± 7.1 kg. All dogs had a body conditioning score between 4/9 and 6/9 [43].

None of the dogs had to be excluded based on the exclusion criteria.

### 3.2. Acclimatization and Trial Duration

The mean acclimatization time was 8.8 ± 4.1 min for time point 1 and 4.9 ± 3.2 min for time point 2. The mean duration of the posturographic measurements was 27.8 ± 10.3 min at time point 1 and 22.8 ± 12.8 min at time point 2.

### 3.3. Center of Pressure (COP) Parameters

#### 3.3.1. Static Condition

**Figure 3:**
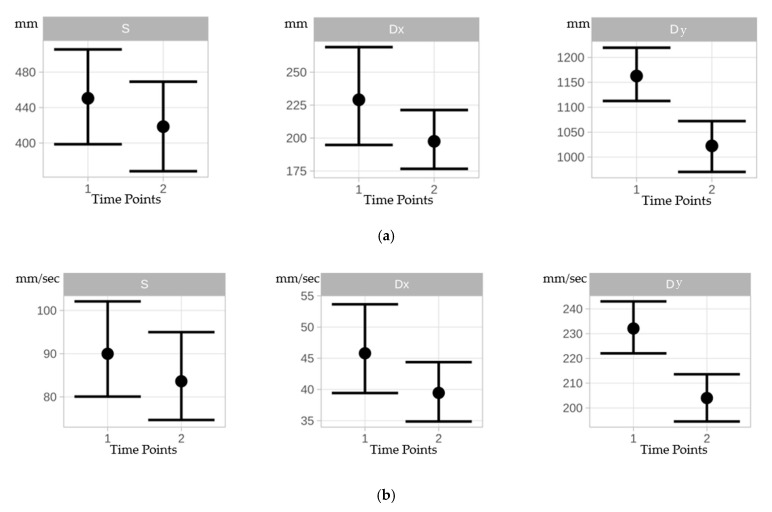

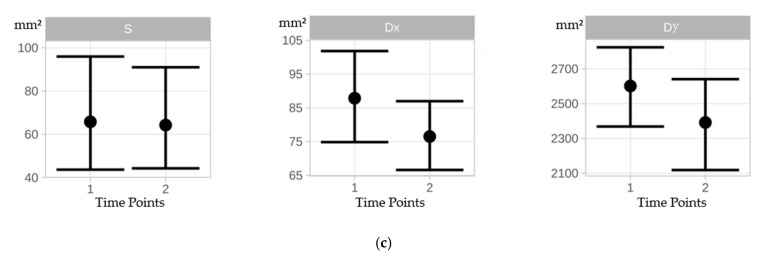
(**a**): Errorbars indicate mean differences between time points 1 and 2 for the area of the 95% COP confidence ellipse for static (S), dynamic x-direction (Dx), and dynamic y-direction (Dy) conditions. (**b**): Errorbars indicating mean differences between time points 1 and 2 for the COP path length for static (S), dynamic x-direction (Dx), and dynamic y-direction (Dy) conditions. (**c**): Errorbars indicating mean differences between time points 1 and 2 for the COP velocity for static (S), dynamic x-direction (Dx), and dynamic y-direction (Dy) conditions.

Under static conditions, all COP parameters (CEA, PL, AV) decreased from time point 1 to time point 2 but did not differ significantly between time points (Table 1, Figure 3). A negligible training effect was observed for mean CEA (Cohen’s d = 0.013) from time point 1 to time point 2. A slight training effect was observed for mean PL (Cohen’s d = 0.205) and mean AV (Cohen’s d = 0.204) from time point 1 to time point 2 (Table 2).

#### 3.3.2. Dynamic Condition

Under dynamic conditions, mean CEA did not differ significantly between time point 1 and time point 2 for both (Dy and Dx) dynamic conditions (*p* = 0.180). Mean PL shortened significantly (Dy: *p* = 0.000, Dx: *p* = 0.045), and mean AV slowed down significantly (Dy: *p* = 0.000, Dx: *p* = 0.043) from time point 1 to time point 2 for both dynamic conditions (Table 1, Figure 3). A negligible training effect was observed from time point 1 to time point 2 for mean CEA (Cohen’s d: Dy/Dx = 0.180) for both dynamic conditions. Slight and moderate training effects were observed from time point 1 to time point 2 for mean PL (Cohen’s d: d = 0.772; dx = 0.265) and mean AV (Cohen’s d: d = 0.783; dx = 0.267) for both dynamic conditions (Table 2).

#### 3.3.3. Comparison between Static and Dynamic Conditions

At both time points, mean CEA was significantly higher under the Dy-condition compared to the static and Dx-condition and significantly higher under the Dx-condition compared to the static condition (*p*-value < 0.0001). For both time points, PL and AV were significantly higher under the Dy-condition compared to the static and the Dx-condition (*p*-value < 0.0001). Significant decreases could be shown for PL and AV, respectively, at the static condition in comparison with the Dx-condition (*p*-value < 0.0001) (Table 3).

## 4. Discussion

In the present study, we investigated the reliability of COP analysis in healthy dogs on a modified motorized Posturomed balance platform under static and dynamic conditions. We hypothesized that the balance ability of healthy dogs can be measured reliably with high reproducibility on this modified Posturomed platform. This hypothesis could only be partially confirmed, as static posturography proved to be reliable when comparing postural analysis at two time points 7 to 14 days apart in a heterogeneously aged group of healthy dogs consisting of different breeds. However, under both dynamic conditions, our study indicated a training effect.

Previous studies showed that center of pressure variability can be reliably measured in healthy dogs and dogs with lameness standing statically on stance analyzers [35,36,41,44]. Similarly, reliability was established in healthy dogs and dogs with ataxia walking on experimental treadmills and walkways for COP analysis without any external perturbation [33,34,36,37].

Our results indicate that the three COP parameters could be reliably measured in healthy dogs at two time points 7 to 14 days apart. This is consistent with a previous study investigating the intersession reliability of healthy dogs standing on a Tekscan walkway (Tekscan Inc, Norwood, MA, United States) [41]. In contrast to this walkway, which does not move at all, the unlocked free-swinging Posturomed balance platform allows, even under static conditions, movements at a horizontal level due to the dampening elements.

Recently, balance response was measured in dogs exposed to combined rotational, eccentrical, and inclinational movements while standing on a motorized platform (Imoove-vet^®^ platform, Allcare Innovations, Bourg les Valence, France) [42]. The authors showed that the Imoove-vet^®^-induced external perturbations significantly affected COP parameters in healthy dogs. However, test-retest reliability was not evaluated in this study.

In contrast to the dogs balancing on the Imoove-vet^®^ platform, the dynamic conditions were well accepted by all dogs balancing on the modified Posturomed platform. While the Posturomed platform solely induces regular motions in a horizontal plane, the Imoove-vet^®^ platform also induces movements in inclined levels. This may indicate that the monoplanar horizontal movements induced by the dynamized modified Posturomed platform require less severe counter-movements to maintain balance during induced perturbations compared to the Imoove-vet^®^ platform. Thus, this type of platform may be more suitable for the balance assessment of dogs with orthopedic or neurologic diseases, as controlled posture can be more easily maintained. On the other hand, although the monoplanar movements have a higher acceptance rate, these perturbations may be less effective for postural and muscular training compared to multiplanar rotational perturbations. Nevertheless, the postural challenge can be raised by an increase in the deflection frequency of the Posturomed or by combination with additional balancing tasks. However, further comparative study between the Imoove-vet^®^ and the modified Posturomed platform would be required to elucidate their role during balance assessment in these patients. It is important to take into consideration that the FDM platform employed for balance analysis in our study and as well in the previous Imoove-vet^®^ study, has not been validated yet for COP assessment in dogs.

Our study showed that under both dynamic conditions, the COP 95% confidence ellipse area was the only reliable COP parameter. This differs from several human balance studies with and without external perturbations, showing that path length and velocity were most reliable [24,25,45].

In our study, COP path length and velocity had decreased from the first to the second time point, indicating a slight training effect. Nevertheless, although this was not observed under static conditions, individual variations, or habituation to the study environment, the observer and the Posturomed may have also affected the results. This finding is consistent with previous observations in human studies showing minor to significant training effects on Posturomed and other balance platforms under dynamic conditions in not only healthy adults but also elderly people [6,26,46]. Superior training of the reaction time and the sensomotoric system under dynamic compared to static conditions provides a possible explanation for this observation [47].

In our study, balancing on the static platform was only minimally challenging for healthy dogs compared to the two dynamic conditions. Thus, static Posturomed balance tests may provide an excellent option to assess dogs with more severe neurologic or orthopedic diseases. We observed that the balance of the dogs was most challenged when the dogs were standing with all four limbs on the platform in a perpendicular direction to the deflection mechanism. When standing with only the forelimbs on the platform opposing the deflection mechanism, COP parameters indicate that the dogs were significantly more stable compared to the Dy position. It remains unclear whether this was primarily due to the placement of the hindlimbs on firm ground or the change in perturbation direction. In humans, perturbation direction has been shown to significantly affect walking instability [48]. Unfortunately, due to the size of the dogs, the narrow longitudinal shape of the modified prototype platform did not allow for the placement of the dogs with all four limbs in the Dx position on the platform to investigate the effect of perturbation direction in our study [42].

There are several limitations to this study. First, dogs of different breeds and with varying body conformation were participating in this study. Although we only enrolled dogs with ideal to slightly above ideal body conditioning scores in this study, we expect that the heterogenicity of our study population affected COP parameters. We can also not exclude that this heterogenicity may have also affected reliability. Thus, further studies with larger numbers are needed to establish concise reference values for balance analysis on this modified Posturomed platform. Additionally, an extensive COP database is needed, taking into account dog breed, body condition, age, training, and health status, to allow for the detection of balance deficits at an early stage during routine balance assessment. Another limitation is the relatively small sample size in our study. In previous veterinary posturographic studies, sample sizes ranged from 6 to 40 animals, with the majority of studies working with group sizes below 30 animals. Thus, our sample size of 20 dogs was deemed appropriate for this very first study. Based on the results of this study, the Posturomed platform can be employed for balance assessment and monitoring of therapeutic success or disease progression in individual healthy dogs, although a training effect needs to be taken into account. In this study, we only compared COP parameters documented during two visits 7 to 14 days apart. Future studies are needed to investigate whether this training effect is still present after two evaluations or subsides.

It was beyond the scope of this study to examine the effect of differing deflection rates on COP parameters and the ability to compensate for these perturbations. We solely investigated dynamic conditions with a frequency of 1.2 horizontal deflections per second. We selected this frequency as preliminary studies showed that healthy dogs with a body weight between 10 and 40 kg were able to adapt quickly to this deflection rate. Moreover, this deflection rate did not trigger excessive compensation stepping. Nevertheless, compensation steps could be observed in many dogs as a reaction to the movement of the balance platform under dynamic conditions. This phenomenon has also been observed in human medicine because the neural and neuromuscular systems prefer stable equilibrium conditions, and a compensation step acts as a posture-stabilizing mechanism [49]. Based on pretrials with differing calibration weights (5–70 kg) on the modified Posturomed platform, minor changes in deflection rate depending on the body weight of the dog have to be taken into consideration. Similarly, we expect that the dog’s body weight may also affect the extent of the dampened motions induced by the unlocked free swinging of the platform under static conditions. However, previous human studies indicate that this was not clinically relevant, as the changes could only be observed with weights positioned on the platform and not with people standing on it [30,46].

For the purpose of this study, we opted to modify the original Posturomed platform with a continuous deflection mechanism, as preliminary trials clearly showed that the dogs consistently reacted with a change in body position and compensation steps when the trigger was manually released. This appeared to be primarily associated with minor movements of the person releasing the trigger and the sound of the trigger release and did not allow for adequate habituation of the dog to the platform. Potentially, a deflection mechanism inducing sudden irregular multidirectional perturbations may allow for more relevant balance assessment for everyday situations (e.g., abrupt speed changes in cars or buses) compared to the continuous regular unidirectional perturbation induced by the current deflection mechanism. In previous human studies, anticipation did not affect susceptibility to walking balance perturbations induced in young adults. However, perturbation direction significantly affected walking instability in this study [48]. Further studies are needed to compare the suitability of different deflection mechanisms for balance assessment depending on health status, fitness, and sport.

## 5. Conclusions

In conclusion, a balance assessment on this modified Posturomed platform was feasible for healthy dogs. Based on the results of this study, COP parameters can be reliably obtained in healthy dogs under static conditions on a modified Posturomed platform. For balance assessment under both dynamic conditions, a mild training effect has to be taken into consideration. The 95% COP confidence ellipse area was the most reliable parameter in healthy dogs and was suitable under static and dynamic conditions. Further studies are needed to investigate Posturomed-based balance analysis in dogs with neurologic and orthopedic diseases.

## Figures and Tables

**Table 1:** Center of pressure (COP) parameters under dynamic and static conditions: Mean ± SD of the area of the 95% COP confidence ellipse, length of COP path, and average COP.

Condition	COP Parameter	Time Point 1	Time Point 2	*p*-Value
Static	Area (mm^2^)	65.8 ± 78.94	64.27 ± 71.7	0.920
	Length (mm)	450.47 ± 181.54	418.55 ± 179.3	0.118
	Velocity (mm/s)	89.98 ± 36.34	83.62 ± 35.83	0.119
Dynamic (y-direction)	Area (mm^2^)	2601.28 ± 713.01	2391.67 ± 699.57	0.180
	Length (mm)	1162.73 ± 191.54	1022.47 ± 171.28	0.000
	Velocity (mm/s)	232.11 ± 38.05	204.01 ± 34.01	0.000
Dynamic (x-direction)	Area (mm^2^)	87.83 ± 46.5	76.49 ± 32.12	0.180
	Length (mm)	229.04 ± 132.05	197.52 ± 80.23	0.045
	Velocity (mm/s)	45.8 ± 26.33	39.45 ± 16.05	0.043

**Table 2:** Center of pressure (COP) parameters under dynamic and static conditions: Mean ± SD differences between time points 1 (TP 1) and 2 (TP 2) and corresponding effect sizes for the area of the 95% COP confidence ellipse, length of the COP path, and COP velocity.

Condition	COP Parameter	Mean ± SD Difference between TP 1 and 2	Cohen’s d [95% CI]
Static	Area (mm^2^)	1.52 ± 117.62	0.013 [−0.242–0.268]
	Length (mm)	31.92 ± 155.83	0.205 [−0.052–0.464]
	Velocity (mm/s)	6.37 ± 31.17	0.204 [−0.053–0.463]
Dynamic (y-direction)	Area (mm^2^)	209.62 ± 1197.21	0.175 [−0.081–0.433]
	Length (mm)	140.25 ± 181.56	0.772 [0.485–1.068]
	Velocity (mm/s)	28.1 ± 35.91	0.783 [0.495–1.079]
Dynamic (x-direction)	Area (mm^2^)	11.34 ± 64.78	0.175 [−0.081–0.433]
	Length (mm)	31.52 ± 119.08	0.265 [0.006–0.525]
	Velocity (mm/s)	6.35 ± 23.78	0.267 [0.009–0.528]

**Table 3:** Comparison between the three conditions Dy (dynamic y-direction), Dx (dynamic x-direction), and S (static) for the three COP parameters. Ratios between conditions and corresponding *p*-values.

Conditions	COP Parameter	Ratio	*p*-Value
Dy/Dx	Area (mm^2^)	30.843	<0.0001
	Length (mm)	5.397	<0.0001
	Velocity (mm/s)	5.388	<0.0001
Dy/S	Area (mm^2^)	50.223	<0.0001
	Length (mm)	2.630	<0.0001
	Velocity (mm/s)	2.628	<0.0001
Dx/S	Area (mm^2^)	1.628	<0.0001
	Length (mm)	0.487	<0.0001
	Velocity (mm/s)	0.488	<0.0001

## Data Availability

Additional research data including further details on protocols, analytic methods, raw data, processed data, and study material is available upon request to interested researchers.
